# Phosphoproteomic Profiling Identifies PAK4 S474 Phosphorylation Affects Docetaxel Chemosensitivity via Modulation of Microtubule Stabilization in Breast Cancer

**DOI:** 10.3390/biomedicines14071631

**Published:** 2026-07-20

**Authors:** Shiyang Liu, Shuyu Li, Zonghong Lu, Xiaofei Tong, Zhengwei Gui, Meina Sun, Lin Zhang

**Affiliations:** Department of Thyroid and Breast Surgery, Tongji Hospital, Tongji Medical College, Huazhong University of Science and Technology, Wuhan 430000, China

**Keywords:** breast cancer, docetaxel, phosphoproteomics, PAK4, microtubule stabilization

## Abstract

**Background/Objectives**: Docetaxel is a frontline chemotherapeutic agent for breast cancer; however, therapeutic resistance remains a major clinical challenge. Emerging evidence suggests that chemotherapy-induced adaptive phosphorylation events can promote survival signaling and contribute to drug resistance. However, the phosphoproteomic mechanisms underlying docetaxel-induced adaptive responses remain poorly characterized. **Methods**: We performed integrated quantitative proteomic and phosphoproteomic profiling in breast cancer cells following docetaxel exposure. Candidate kinases associated with phosphorylation remodeling were identified and validated using TCGA-BRCA and CPTAC clinical datasets. The functional significance of PAK4 phosphorylation was validated using phosphomimetic and phospho-deficient mutants, pharmacological inhibition, and assessment of microtubule stabilization. **Results**: Integrated phosphoproteomic analysis revealed extensive phosphorylation remodeling following docetaxel treatment and identified PAK4 as a candidate kinase associated with the adaptive response. Analysis of the CPTAC phosphoproteomic dataset showed that phosphorylation of PAK4 at S474 was elevated in breast cancer tissues, increased with tumor stage, and was associated with poorer overall survival. In breast cancer cells, docetaxel induced phosphorylation of PAK4 at S474 without altering total PAK4 expression. Functionally, phosphomimetic PAK4 (S474D) reduced docetaxel sensitivity, whereas phospho-deficient PAK4 (S474A) enhanced drug sensitivity. Pharmacological inhibition of PAK4 using LCH-7749944 significantly enhanced the inhibitory effect of docetaxel on cell viability and increased apoptosis in breast cancer cells. Mechanistically, PAK4 inhibition enhanced docetaxel-induced microtubule stabilization, as evidenced by increased α-tubulin acetylation and accumulation of stabilized microtubule structures. **Conclusions**: Our study demonstrates that docetaxel induces global phosphorylation network reprogramming in breast cancer cells and identifies PAK4 S474 phosphorylation as a key determinant of docetaxel sensitivity. Inhibition of PAK4 enhances microtubule stabilization and improves the efficacy of docetaxel, providing a potential combinatorial strategy to overcome taxane resistance.

## 1. Introduction

Breast cancer remains the leading cause of cancer-related mortality among women worldwide, with increasing incidence and substantial clinical heterogeneity [1]. Although substantial advances have been made in systemic therapies, the long-term prognosis of patients with advanced disease remains suboptimal [2]. Docetaxel, a taxane-class chemotherapeutic agent, is widely used in the treatment of breast cancer due to its ability to stabilize microtubules, thereby inducing mitotic arrest and apoptosis [3,4]. However, both intrinsic and acquired resistance, together with dose-limiting toxicities, continue to limit its clinical efficacy [5].

Chemotherapeutic agents induce a range of adaptive cellular responses—such as autophagy activation, ER stress signaling, metabolic reprogramming, and signaling pathway rewiring—that enable cancer cells to survive under drug stress [6,7]. In particular, protein phosphorylation-mediated signaling reprogramming plays a central role in coordinating cellular responses to chemotherapeutic stress [8]. Chemotherapeutic agents have been shown to induce rapid and dynamic rewiring of kinase signaling networks, which may paradoxically promote survival and contribute to drug resistance. For instance, docetaxel has been reported to modulate tumor-associated signaling through phosphorylation of Akt, thereby influencing therapeutic response in cancer cells [9]. However, a systematic and global understanding of docetaxel-induced phosphorylation remodeling in breast cancer cells remains largely incomplete.

p21-activated kinase 4 (PAK4), a serine/threonine kinase downstream of Rho GTPases, is a critical regulator of cytoskeletal organization, cell motility, and survival signaling [10]. Aberrant expression and activation of PAK4 has been implicated in tumor progression and poor clinical outcomes across multiple cancer types [11]. Nevertheless, whether PAK4 participates in chemotherapy-induced adaptive signaling and contributes to docetaxel resistance in breast cancer remains unclear.

In this study, we performed integrated quantitative proteomic and phosphoproteomic analyses using liquid chromatography–tandem mass spectrometry (LC–MS/MS) to systematically characterize docetaxel-induced signaling alterations in breast cancer cells. We identified p21-activated kinase 4 (PAK4) phosphorylation at S474 as a key adaptive signaling event and demonstrated its functional role in regulating the cellular response to docetaxel. Inhibition of PAK4 enhances docetaxel-induced cytotoxicity by promoting microtubule stabilization, highlighting a previously unrecognized mechanism by which adaptive PAK4 phosphorylation regulates the cellular response to docetaxel.

## 2. Materials and Methods

### 2.1. Cell Culture

HCC1937 (RRID: CVCL_0290), MDA-MB-231 (RRID: CVCL_0062), SKBR3 (RRID: CVCL_0033), MCF-7 (RRID: CVCL_0031) and MDA-MB-468 (RRID: CVCL_0419) human breast cancer cell lines were cultured in DMEM medium (Hyclone, Logan, UT, USA), whereas T-47D (RRID: CVCL_0553) human breast cancer cell line was cultured in RPMI-1640 medium (Hyclone, Logan, UT, USA). All media was supplemented with a 10% fetal bovine serum (FBS) (Invitrogen, Gaithersburg, MD, USA). All the cells were incubated in a humid atmosphere with 5% CO2. The human breast cancer cell lines used in the experiments were obtained from the Shanghai Institute of Cell Biology (Shanghai, China) within the last three years and authenticated using short tandem repeat profiling.

### 2.2. Liquid Chromatography–Tandem Mass Spectrometry Analysis and Bioinformatics Analysis

MDA-MB-231 cells were treated with docetaxel or DMSO for 12 h and subsequently lysed on ice in lysis buffer (8 M urea and 1% Protease Inhibitor Cocktail), and centrifuged at 12,000× *g* at 4 °C for 10 min. Then, the supernatants were collected, digested and labeled in TMT reagent. After the TMT-labeled tryptic peptides were separated with a reverse-phase high-performance liquid chromatography (HPLC) method, samples were desalted via C18 ZipTips (Millipore, Burlington, MA, USA), vacuum-dried, and further enriched for phosphorylation. Then, the peptides were analyzed by tandem mass spectrometry in Q ExactiveTM Plus (Thermo, Bremen, Germany) coupled to an EASY-nLC 1000 UPLC system. The resulting data were processed for peptide identification by database searching using the Maxquant search engine (v.1.5.2.8). Tandem mass spectra were searched against the human uniprot database. Trypsin/P was specified as the cleavage enzyme, allowing up to 4 missing cleavages. Allowed fixed modifications included carbamidomethylation on cysteine, where oxidation on methionine, acetylation on protein N-terminal and phosphorylation on serine, threonine, and tyrosine were set as variable modifications. False discovery rates (FDR) on proteins and phosphorylated peptides were adjusted to less than 1%. Phosphorylation abundance was normalized to the corresponding protein abundance to distinguish phosphorylation-specific regulation from changes in protein expression. Significantly altered phosphorylation sites were identified using a two-tailed Student’s *t*-test (*p* < 0.05) and fold-change thresholds (>1.4 or <1/1.4). Functional enrichment analyses including GO, KEGG, and GSEA were performed using the R package “clusterProfiler”. Protein–protein interaction (PPI) networks were constructed using the STRING database.

### 2.3. Public Datasets Analysis

Different mRNA expression between breast cancer tumor tissue and normal tissue was explored in the TCGA database, with ANOVA being used to analyze the statistical significance. The correlation between the mRNA expression and DFS or OS was analyzed by Cox regression in the TCGA database. The association between gene expression and response to neoadjuvant chemotherapy was analyzed using the ROC Plotter database (https://rocplot.com/, accessed on 10 January 2021), which integrates transcriptomic data from multiple GEO datasets [12]. Patients were stratified into pathological complete response (pCR) and non-pCR groups. Differences in gene expression were assessed using the Mann–Whitney U test. Multiple testing correction was performed using the Benjamini–Hochberg method where applicable. No additional cutoff was applied beyond the default ROC Plotter classification criteria. Phosphoproteomics data of breast cancer were obtained from the Clinical Proteomic Tumor Analysis Consortium (CPTAC) database (https://pdc.cancer.gov/pdc/cptac-pancancer, accessed on 20 January 2021) [13]. Processed and normalized datasets were downloaded from the CPTAC data portal. Samples lacking complete clinical annotation or phosphorylation quantification were excluded.

### 2.4. Plasmid Construction and Transfection

Cells were transfected with plasmids encoding wild-type PAK4 (PAK4-WT), phosphomimetic mutant PAK4-S474D, or phosphorylation-deficient mutant PAK4-S474A using Lipofectamine 3000 reagent (Thermo Fisher Scientific) according to the manufacturer’s instructions. Briefly, cells were seeded in appropriate culture plates and transfected when they reached approximately 70–80% confluence. After 24–48 h of transfection, the expression levels of PAK4 and p-PAK4 were confirmed by Western blotting, and cells were subsequently subjected to docetaxel treatment and functional assays.

### 2.5. Cell Viability Assay

The experimental cells were plated in 96-well plates (2 × 10^5^/mL/well). Docetaxel (5, 20, 35, 50, 75, and 100 ng/mL) and/or LCH-7749944 (5 and 10 μg/mL) were added to the cells to assess the cell viability. The viability of cells was assessed by one-hour incubation with 10% CCK8 (Cell Counting Kit-8) solution at 37 °C. Cells were lysed in DMEM medium and absorbance was determined at 450 nm in a microplate reader. In addition, docetaxel (5, 20, 35, 50, 75, and 100 ng/mL) and/or LCH-7749944 (5 and 10 μg/mL) were added to the cells to assess the cell viability in the same way.

### 2.6. Flow Cytometry

Cells were collected after EDTA-free trypsin reagent digestion, and resuspended in binding buffer after centrifugation. Next, cells were incubated with Annexin V-FITC at 4 °C in a darkroom for 15 min, and stained by propidium iodide (PI) at 4 °C in a darkroom for 15 min. At last, the apoptotic rates were detected by flow cytometry (BD Biosciences, Franklin Lakes, NJ, USA).

### 2.7. Western Blotting

Cells were collected and lysed in RIPA buffer with protease inhibitors, incubated on ice for 15 min and cleared by centrifugation at 12,000 rpm at 4 °C for 15 min. The protein was separated by SDS–polyacrylamide gel electrophoresis, transferred to polyvinylidene difluoride membrane and incubated with antibodies against GAPDH from Sigma (St. Louis, MO, USA); p-PAK4 (S474) from Santa Cruz (Dallas, TX, USA); PAK4 from Santa Cruz (Dallas, TX, USA); acetyl-α-tubulin from Abcam (Waltham, MA, USA); α-tubulin from Abcam (Waltham, MA, USA) for overnight at 4 °C.

### 2.8. Immunofluorescence

Cells were washed with precooling PBS and incubated with 4% paraformaldehyde and 0.05% Triton X-100 at 4 °C for 15 min. Then cells were incubated with goat serum with antibodies against acetyl-α-tubulin from Abcam (Waltham, MA, USA); α-tubulin from Abcam (Waltham, MA, USA); Ki-67 from Abcam (Waltham, MA, USA) at 4 °C overnight. Next, cells were incubated with a Cy3 conjugated anti-mouse secondary antibody or FITC conjugated anti-rabbit secondary (Servicebio, Wuhan, China), and visualized by confocal fluorescence microscopy (Olympus, Tokyo, Japan) or inverted fluorescence Microscope (Olympus, Tokyo, Japan).

### 2.9. Statistical Analysis

Statistical analyses were performed using R software (version 4.3.2, R Foundation for Statistical Computing, Vienna, Austria) and GraphPad Prism (version 9.5.1, GraphPad Software, Boston, MA, USA). Data are presented as the mean ± SD from at least three independent experiments. Comparisons between two groups were performed using two-tailed Student’s *t*-test. Comparisons among three or more groups were analyzed using one-way ANOVA followed by Tukey’s multiple comparisons test. For experiments involving two independent variables, two-way ANOVA followed by multiple comparisons was used. Pearson correlation analysis was performed to evaluate the association between PAK4 phosphorylation levels and cellular sensitivity to docetaxel across different breast cancer cell lines. Pearson χ^2^ test or Fisher’s exact test was used to analyze categorical variables. Survival analyses were performed using the Kaplan–Meier method with the log-rank test, and the optimal cut-off values were determined using the surv_cutpoint function in the survminer package. A two-sided *p* < 0.05 was considered statistically significant.

## 3. Results

### 3.1. Phosphoproteomic and Proteomic Landscape of Docetaxel-Treated MDA-MB-231 Cells

To systematically characterize the molecular alterations induced by docetaxel, quantitative proteomic and phosphoproteomic analyses were performed in MDA-MB-231 cells treated with docetaxel for 12 h. In total, 6487 proteins were identified, and 11,445 phosphorylation sites corresponding to 3520 proteins were detected, with 8502 of these sites corresponding to 2987 proteins being quantified (Supplementary Tables S1 and S2; Supplementary Figure S1).

**Figure 1:**
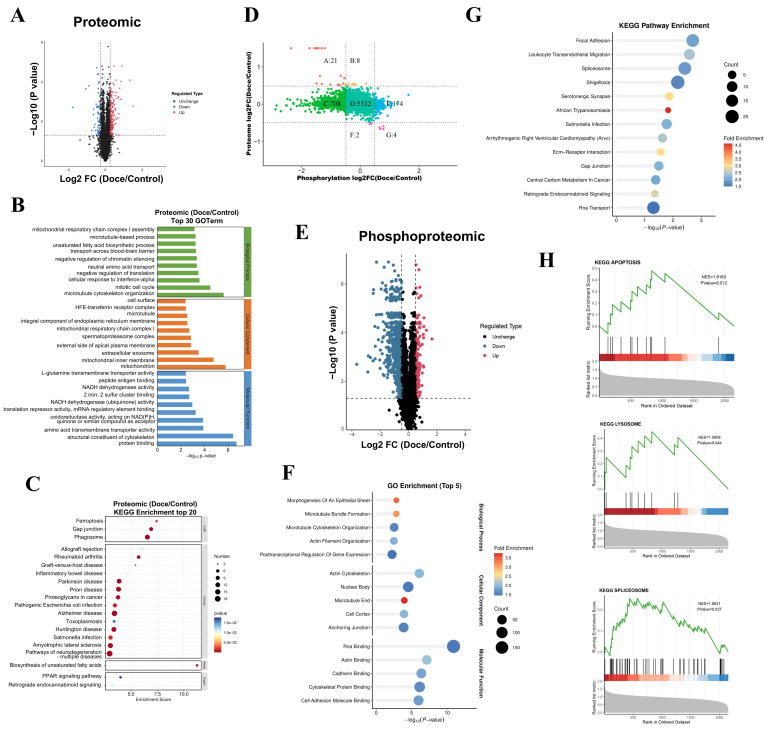
Phosphoproteomic and proteomic landscape of MDA-MB-231 cells treated with docetaxel. (**A**) Volcano plot showing differentially expressed proteins between the docetaxel-treated and control groups. (**B**,**C**) Top 30 GO term enrichment and Top 20 KEGG pathway enrichment analyses of the differentially expressed proteins. (**D**) Nine-quadrant plot illustrating the correlation between protein expression (log2 FC (Doce/Control), y-axis) and phosphorylation levels (log2 FC (Doce/Control), x-axis). Different colors indicate phosphorylation sites located in different quadrants. Quadrants A-H represent different regulatory patterns. (**E**) Volcano plot representing significantly upregulated (red) and downregulated (blue) phosphorylation sites. (**F**,**G**) Dot plots illustrating the significantly enriched GO terms and KEGG pathways derived from the differentially phosphorylated proteins. (**H**) Gene Set Enrichment Analysis (GSEA) plots showing significant enrichment in apoptosis, lysosome and spliceosome pathways following docetaxel treatment. Doce, docetaxel; BP, biological process; CC, cellular component; MF, molecular function; NES, normalized enrichment score.

Global proteomic analysis revealed a distinct expression landscape between control and docetaxel-treated cells, as demonstrated by volcano plot analysis showing a subset of significantly upregulated and downregulated proteins upon treatment (Figure 1A). To further explore the biological implications of these changes, Gene Ontology (GO) enrichment analysis indicated that differentially expressed proteins were mainly associated with processes related to cellular organization and stress-responsive pathways (Figure 1B). Consistently, KEGG pathway analysis highlighted significant enrichment in multiple cancer-relevant signaling and metabolic pathways, suggesting that docetaxel broadly reshapes cellular proteostasis and functional networks (Figure 1C).

Following normalization of phosphorylation levels to corresponding protein abundance, 6249 phosphorylation sites were retained for downstream analysis (Figure 1D). Differential expression analysis revealed that 163 phosphorylation sites in 110 proteins were significantly upregulated, whereas 709 sites in 475 proteins were significantly downregulated in docetaxel-treated cells compared with controls (Figure 1E). GO enrichment analysis of differentially phosphorylated proteins demonstrated that the affected sites were predominantly enriched in biological processes related to cytoskeletal organization, cell adhesion, and intracellular transport, as well as RNA metabolism-associated functions (Figure 1F). Complementary KEGG pathway analysis further confirmed that focal adhesion, ECM–receptor interaction, spliceosome, and gap junction pathways were among the most significantly enriched signaling modules (Figure 1G). Gene set enrichment analysis (GSEA) revealed that docetaxel treatment significantly altered pathways involved in apoptosis, lysosomal function and the spliceosome (Figure 1H). These results indicate that docetaxel induces coordinated phosphorylation remodeling in both structural and post-transcriptional regulatory networks.

### 3.2. Identification of PAK4 as a Candidate Kinase Associated with Docetaxel Resistance

**Figure 2:**
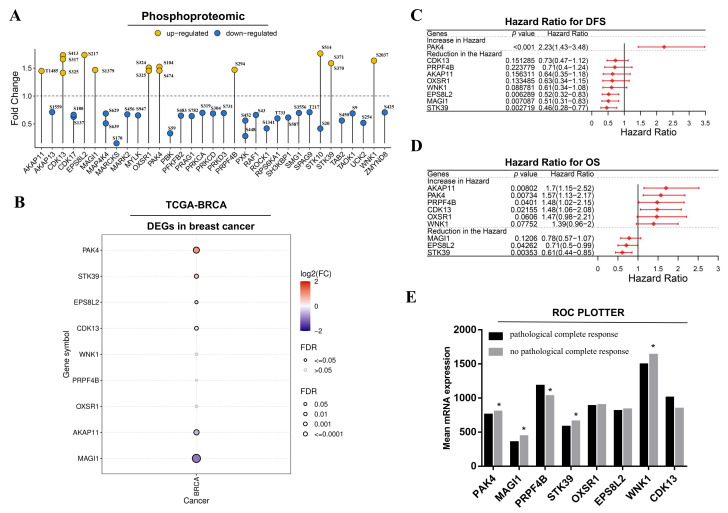
Identification of upregulated kinases associated with docetaxel resistance. (**A**) Dumbbell plot showing differential phosphorylation changes in kinase proteins identified from phosphoproteomic analysis of docetaxel-treated MDA-MB-231 cells. Kinases with increased (yellow) or decreased (blue) phosphorylation are shown. (**B**) Bubble plot illustrating mRNA expression levels of candidate kinases in TCGA-BRCA. Color intensity represents log2 (fold change), and circle size represents statistical significance (FDR). (**C**,**D**) Forest plots showing hazard ratios (HR) of candidate kinase mRNA expression for disease-free survival (DFS, (**C**)) and overall survival (OS, (**D**)) based on TCGA data. (**E**) Comparison of mRNA expression of candidate kinases between pCR and non-pCR patients from the ROC Plotter database. Data are presented as mean ± SD. * *p* < 0.05 by two-tailed Student’s *t*-test. DFS, disease-free survival; OS, overall survival; HR, hazard ratio; pCR, pathological complete response.

Given the critical role of protein kinases in signal transduction and drug response, we next analyzed kinases with significant phosphorylation alterations following docetaxel treatment. By integrating phosphorylation changes with corresponding protein abundance, we prioritized kinases showing increased phosphorylation without substantial changes in total protein levels. Nine candidate kinases were identified, including PAK4, MAGI1, PRPF4B, STK39, OXSR1, EPS8L2, WNK1, CDK13, and AKAP11 (Figure 2A).

To assess their clinical relevance, we analyzed mRNA expression profiles in the TCGA-BRCA dataset. Among these candidates, PAK4 was the most significantly upregulated kinase in breast cancer tissues compared to normal tissues (Figure 2B) and the only kinase consistently associated with both disease-free survival (DFS) and overall survival (OS) (Figure 2C,D), indicating its potential role in tumor progression.

To further evaluate the association with therapeutic response, we analyzed a cohort of breast cancer patients receiving taxane-based neoadjuvant chemotherapy. The mRNA expression levels of PAK4, MAGI1, STK39, and WNK1 were significantly higher in non-pCR patients compared to pCR patients (Figure 2E). Taken together, these results identified PAK4 as the most promising candidate kinase associated with docetaxel response and survival outcomes, and it was selected for further functional investigation.

### 3.3. PAK4 Phosphorylation at S474 Is Associated with Poor Prognosis in Breast Cancer

**Figure 3:**
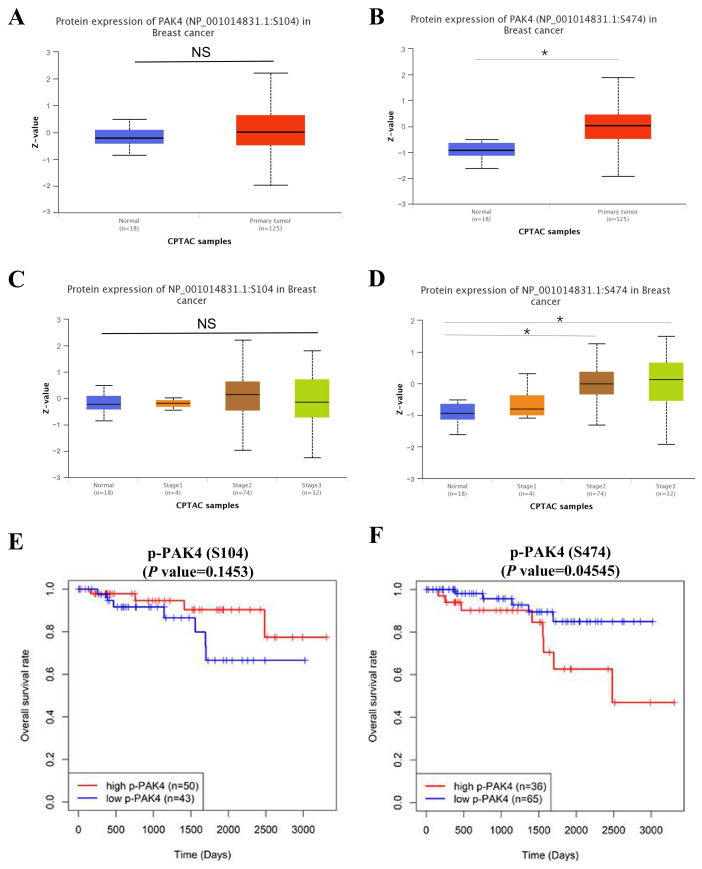
Elevated PAK4 phosphorylation at S474 predicts poor prognosis in breast cancer using the CPTAC cohort. (**A**,**B**) Protein expression levels (Z-values) of phosphorylated PAK4 at residues S104 (**A**) and S474 (**B**) in normal tissues compared to primary breast cancer tumors from the CPTAC dataset. (**C**,**D**) Expression levels of p-PAK4 S104 (**C**) and S474 (**D**) across different clinical stages (Stage 1, 2, and 3) of breast cancer. (**E**,**F**) Kaplan–Meier survival curves showing the correlation between the phosphorylation levels of PAK4 at S104 (**E**) and S474 (**F**) and the overall survival (OS) of breast cancer patients. NS, not significant. * *p* < 0.05. Statistical significance was determined by one-way ANOVA or Student’s *t*-test.

To further investigate the clinical significance of PAK4 phosphorylation, we analyzed phosphoproteomic data from the CPTAC cohort. Phosphorylation of PAK4 at S474 was significantly elevated in primary breast cancer tissues compared to normal tissues, whereas phosphorylation at S104 did not show a comparable increase (Figure 3A,B). In addition, phosphorylation at S474 exhibited a progressive increase across clinical stages, while S104 remained relatively unchanged (Figure 3C,D).

Kaplan–Meier survival analysis demonstrated that patients with high levels of PAK4 phosphorylation at S474 had significantly poorer overall survival, whereas phosphorylation at S104 was not associated with patient outcomes (Figure 3E,F). These results indicate that phosphorylation of PAK4 at S474, but not S104, is clinically relevant and may serve as a prognostic biomarker in breast cancer.

Furthermore, phosphoproteomic network analysis revealed that phosphorylated PAK4 is functionally associated with key oncogenic signaling pathways, including focal adhesion, ErbB signaling, and RAS signaling pathways (Figure S2), suggesting its involvement in tumor-promoting signaling networks.

### 3.4. Docetaxel-Induced PAK4 S474 Phosphorylation Reduced Sensitivity to Docetaxel in Breast Cancer Cells

**Figure 4:**
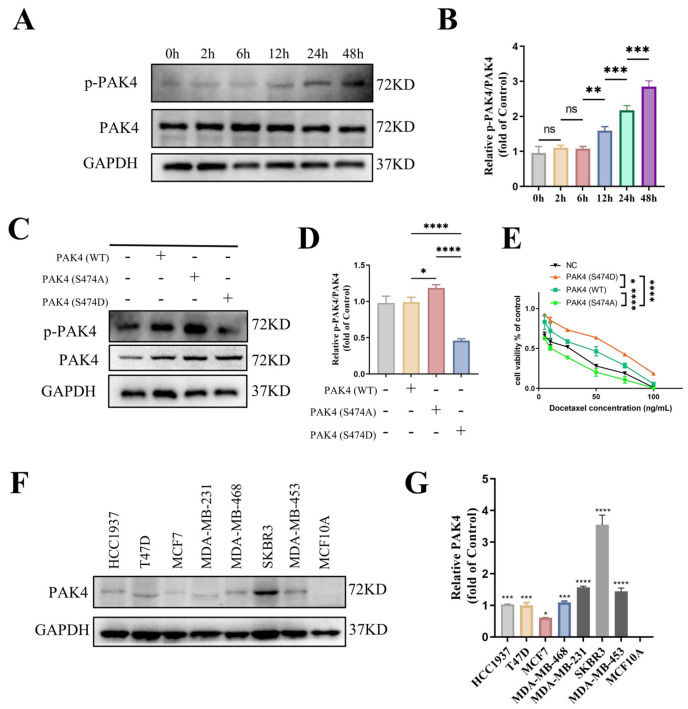
Functional validation of PAK4 S474 phosphorylation in regulating docetaxel sensitivity in breast cancer cells. (**A**,**B**) Western blot analysis and quantification of PAK4 phosphorylation at S474 in breast cancer cells treated with docetaxel at the indicated time points (0, 2, 6, 12, 24, and 48 h). Total PAK4 and GAPDH served as controls. (**C**,**D**) Western blot analysis and quantitative evaluation of p-PAK4 and PAK4 expression in cells transfected with wild-type PAK4 (WT), phospho-deficient (S474A), or phosphomimetic (S474D) PAK4 plasmids. (**E**) Cell viability curves of cells expressing different PAK4 mutants under various concentrations of docetaxel treatment. (**F**,**G**) Baseline protein expression of PAK4 across multiple breast cancer cell lines (HCC1937, T47D, MCF7, MDA-MB-231, MDA-MB-468, SKBR3, MDA-MB-453) and the normal mammary epithelial cell line MCF10A, assessed by Western blot and quantified relative to MCF10A. Data are presented as mean ± SD. Statistical significance was determined by one-way ANOVA or two-way ANOVA. * *p* < 0.05, ** *p* < 0.01, *** *p* < 0.001, **** *p* < 0.0001; ns, not significant.

To investigate whether PAK4 activation contributes to the adaptive response of breast cancer cells to docetaxel treatment, we first examined the dynamic changes in PAK4 phosphorylation following docetaxel exposure. Time-course analysis demonstrated that docetaxel treatment progressively increased phosphorylation of PAK4 at S474 from 12 h to 48 h, whereas total PAK4 protein levels remained unchanged (Figure 4A,B), indicating that docetaxel regulates PAK4 activity primarily through post-translational modification.

To further determine whether PAK4 phosphorylation is associated with docetaxel response, we analyzed the correlation in T47D, MCF7, MDA-MB-231 and SKBR3 cell lines. The relative levels of docetaxel-induced p-PAK4 (S474)/PAK4 showed a positive correlation trend with cell viability after 50 ng/mL docetaxel treatment across four breast cancer cell lines (Figure S3A–D), suggesting that enhanced PAK4 activation is associated with reduced docetaxel sensitivity.

To establish the functional significance of PAK4 S474 phosphorylation, MDA-MB-231 cells were transfected with WT PAK4, phospho-deficient PAK4-S474A, or phosphomimetic PAK4-S474D mutants. Compared with WT PAK4, S474D expression significantly decreased docetaxel sensitivity, whereas S474A expression enhanced docetaxel sensitivity (Figure 4C–E), demonstrating that PAK4 S474 phosphorylation functionally regulates docetaxel response.

Among the examined breast cancer cell lines, MDA-MB-231 and SKBR3 cells showed relatively higher endogenous PAK4 expression compared with MCF10A cells and were selected for subsequent experiments (Figure 4F,G). Furthermore, in MCF7 cells with low endogenous PAK4 expression, overexpression of PAK4-S474D significantly reduced docetaxel sensitivity compared with WT PAK4 (Figure S4A–C), further confirming the role of PAK4 S474 phosphorylation in promoting docetaxel resistance.

### 3.5. Pharmacological Inhibition of PAK4 Enhances Docetaxel Sensitivity in Breast Cancer Cells

**Figure 5:**
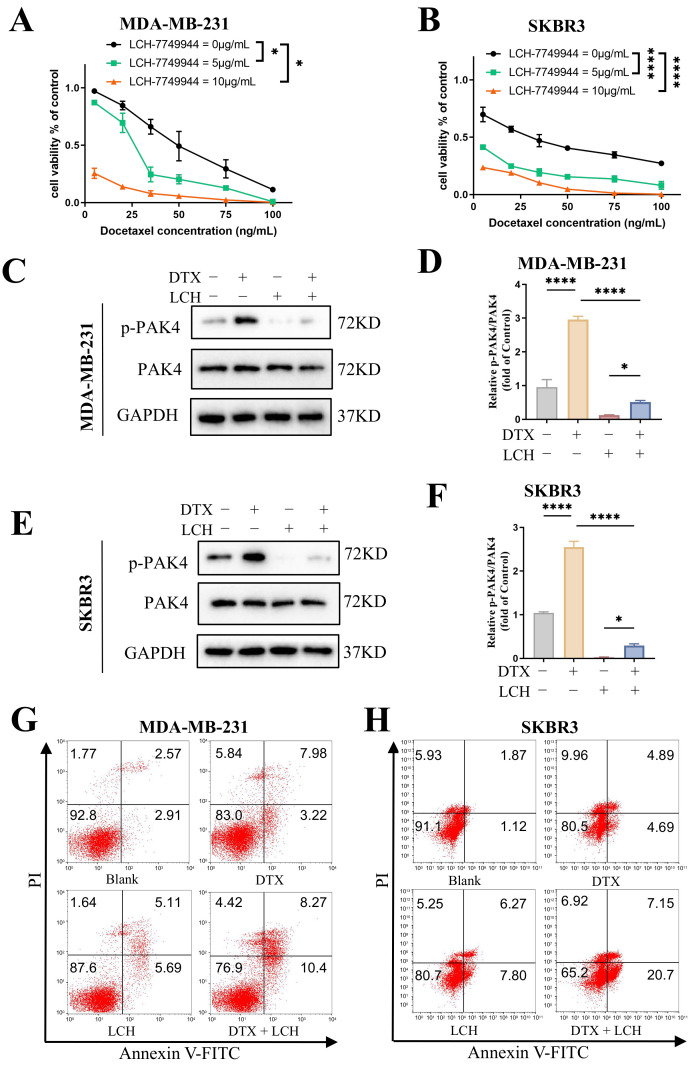
Inhibition of PAK4 activation by LCH-7749944 enhances the sensitivity of breast cancer cells to docetaxel. (**A**,**B**) Effect of the PAK4 inhibitor LCH-7749944 (0, 5, and 10 µg/mL) alone or in combination with docetaxel on the cell viability of MDA-MB-231 (**A**) and SKBR3 (**B**) cells. (**C**–**F**) Western blot analysis of p-PAK4 and PAK4 levels (**C**,**E**) and their quantitative ratios (**D**,**F**) in MDA-MB-231 (**C**,**D**) and SKBR3 (**E**,**F**) cells following treatment with docetaxel (DTX) and/or LCH-7749944 (LCH). (G-H) Representative flow cytometry plots (Annexin V-FITC/PI staining) showing apoptosis in MDA-MB-231 (**G**) and SKBR3 (**H**) cells subjected to the indicated treatments. Data are presented as mean ± SD. Statistical significance was determined by one-way ANOVA or two-way ANOVA. * *p* < 0.05, **** *p* < 0.0001.

To further validate the therapeutic relevance of PAK4 activation in docetaxel response, we examined whether pharmacological inhibition of PAK4 by selective PAK4 inhibitor LCH-7749944 could enhance the efficacy of docetaxel [14]. The results showed that treatment with LCH-7749944 significantly enhanced the inhibitory effects of docetaxel, resulting in a further reduction in cell viability in both MDA-MB-231 and SKBR3 cells (Figure 5A,B). Western blot analysis showed that LCH-7749944 markedly suppressed docetaxel-induced phosphorylation of PAK4 at S474, while total PAK4 protein levels remained unchanged in both cell lines (Figure 5C–F), indicating that LCH-7749944 primarily inhibits PAK4 activation through modulation of phosphorylation rather than altering PAK4 expression. Consistently, flow cytometric analysis demonstrated that combined treatment with LCH-7749944 and docetaxel significantly increased apoptotic cell populations compared with either agent alone in both MDA-MB-231 and SKBR3 cells (Figure 5G,H). These findings demonstrate that pharmacological inhibition of PAK4 activation enhances docetaxel-induced cytotoxicity and supports PAK4 as a potential therapeutic target for overcoming docetaxel resistance in breast cancer.

### 3.6. PAK4 Inhibition Enhances Docetaxel-Induced Microtubule Stabilization

**Figure 6:**
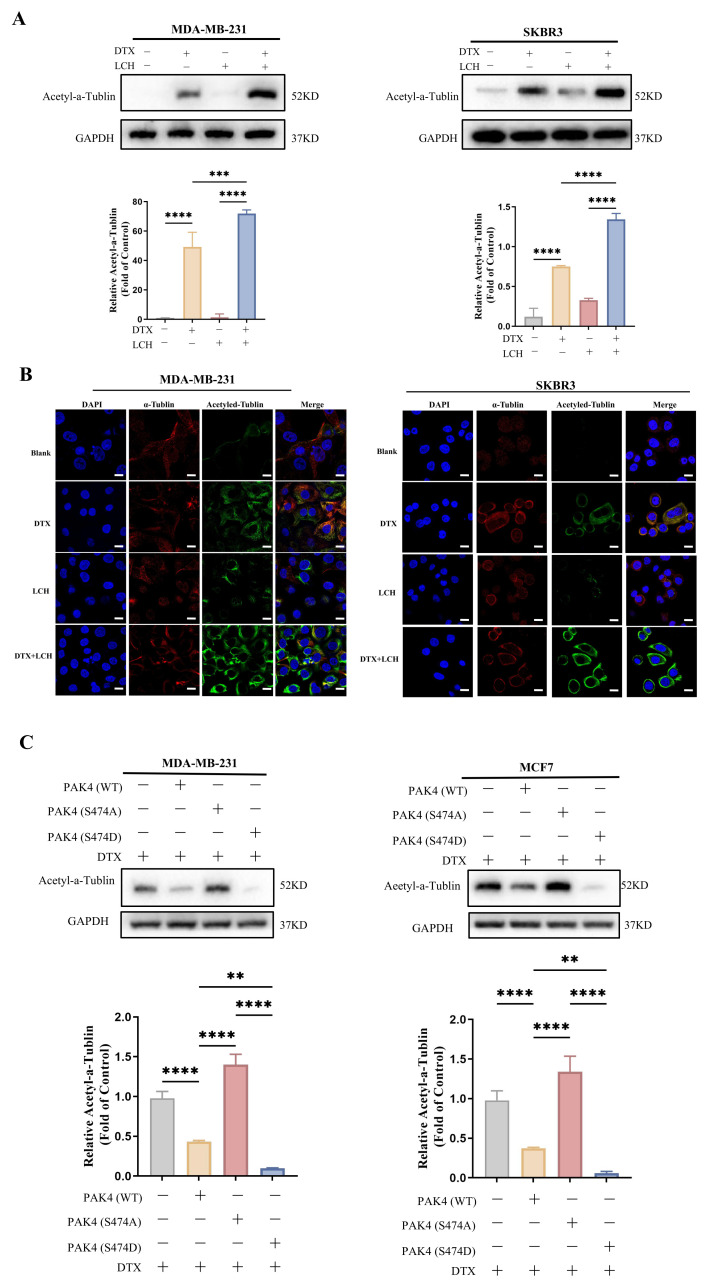
PAK4 phosphorylation negatively regulates docetaxel-induced microtubule stabilization. (**A**) Western blot analysis and quantification of acetylated α-tubulin (Acetyl-α-Tubulin) expression in MDA-MB-231 and SKBR3 cells treated with LCH-7749944 (LCH) and/or docetaxel (DTX). (**B**) Confocal microscopic visualization of microtubule stabilization. Cells were incubated with DAPI (blue), immunostained for acetylated α-tubulin (green) and α-tubulin (red) in MDA-MB-231 and SKBR3 cells treated with LCH-7749944 and/or docetaxel. Scale bars, 5 μm. (**C**) Western blot analysis and quantification of acetylated α-tubulin expression in MDA-MB-231 and MCF7 cells transfected with wild-type (WT), phospho-deficient (S474A), or phosphomimetic (S474D) PAK4 plasmids under docetaxel treatment. Data are presented as mean ± SD. Statistical significance was determined by one-way ANOVA. ** *p* < 0.01, *** *p* < 0.001, **** *p* < 0.0001.

To further investigate the molecular mechanism underlying PAK4-mediated docetaxel resistance, we examined whether PAK4 activation influences the microtubule-stabilizing effect of docetaxel. Since stabilization of microtubules is the primary mechanism of action of taxanes [15], and acetylation of α-tubulin is widely used as a marker of stable microtubules, we assessed acetylated α-tubulin levels following different treatments. Western blot analysis showed that combined treatment with LCH-7749944 and docetaxel significantly increased acetylated α-tubulin expression compared with docetaxel treatment alone in both MDA-MB-231 and SKBR3 cells (Figure 6A). Consistently, confocal microscopy demonstrated enhanced accumulation of acetylated α-tubulin and increased formation of filamentous microtubule structures following combined treatment, indicating enhanced microtubule stabilization (Figure 6B).

To further determine whether PAK4 phosphorylation directly regulates microtubule stabilization, cells expressing different PAK4 mutants were examined under docetaxel treatment. Expression of the phosphomimetic S474D mutant markedly reduced docetaxel-induced α-tubulin acetylation, whereas the phosphorylation-deficient S474A mutant enhanced α-tubulin acetylation compared with WT PAK4 (Figure 6C). Together, these findings demonstrate that PAK4 S474 phosphorylation negatively regulates docetaxel-induced microtubule stabilization, thereby attenuating the therapeutic efficacy of docetaxel.

## 4. Discussion

In the present study, we systematically characterized the protein alterations and phosphoproteomic alterations induced by docetaxel in breast cancer cells using integrated proteomic and phosphoproteomic analyses. By combining phosphoproteomic profiling, clinical database analyses, correlation analyses across multiple breast cancer cell lines, genetic manipulation of PAK4 phosphorylation, and pharmacological inhibition, we demonstrate that docetaxel induces adaptive phosphorylation of PAK4 at S474, which attenuates the cytotoxic efficacy of docetaxel. Mechanistically, inhibition of PAK4 restores docetaxel-induced microtubule stabilization, thereby enhancing apoptosis and drug sensitivity. These findings identify PAK4 phosphorylation as a previously unrecognized adaptive phosphorylation event that limits the therapeutic efficacy of docetaxel.

Phosphorylation-dependent signaling plays a central role in cellular adaptation to chemotherapeutic stress. Our phosphoproteomic analysis revealed widespread remodeling of phosphorylation networks upon docetaxel exposure, affecting pathways involved in focal adhesion, ECM–receptor interaction, spliceosome, and gap junction. These pathways have been implicated in the regulation of cell survival, stress responses, and cellular plasticity, suggesting that docetaxel induces a coordinated adaptive signaling reprogramming rather than a purely cytotoxic effect, consistent with previous reports that chemotherapeutic agents can trigger dynamic rewiring of intracellular signaling networks [16,17]. These findings support the concept that chemotherapy not only induces cytotoxicity but also activates compensatory signaling pathways that facilitate cellular adaptation and therapeutic resistance.

Among the kinases exhibiting increased phosphorylation, PAK4 emerged as a key candidate linking phosphoproteomic alterations to clinical outcomes. While previous studies have established that PAK4 is overexpressed in breast cancer and contributes to tumor progression, invasion, and metastasis [18,19,20,21], our findings extend these observations by identifying phosphorylation of PAK4 at S474 as a clinically relevant event. Analysis of the CPTAC phosphoproteomic dataset further showed that phosphorylation of PAK4 at S474 was elevated in breast cancer tissues, increased with tumor stage, and was associated with poorer overall survival. Importantly, our functional studies further demonstrated that phosphorylation of PAK4 at S474 functionally regulates the cellular response to docetaxel. Across multiple breast cancer cell lines, higher levels of PAK4 phosphorylation were positively correlated with cell viability following docetaxel treatment, while both genetic manipulation of PAK4 phosphorylation and pharmacological inhibition consistently enhanced docetaxel sensitivity. Together, these findings indicate that PAK4 phosphorylation is not merely a prognostic marker but also a functional mediator of adaptive responses to docetaxel.

Drug-induced adaptive kinase activation has emerged as an important mechanism by which cancer cells survive therapeutic stress. Previous studies have shown that docetaxel treatment can activate several pro-survival signaling pathways, including Akt phosphorylation, HSP27 activation, and TLR4–MyD88 signaling, thereby attenuating therapeutic efficacy [9,22,23]. Our findings expand this concept by identifying PAK4 phosphorylation at S474 as another adaptive signaling event induced by docetaxel. Although PAK4 has previously been proposed as a therapeutic target in endocrine therapy and chemotherapy [18,24], our study further identifies adaptive phosphorylation of PAK4 at S474 as a mechanistic basis underlying its regulation of docetaxel sensitivity.

Mechanistically, our data indicate that PAK4 inhibition enhances acetylation of α-tubulin and the microtubule-stabilizing effects of docetaxel. Microtubule dynamics are a primary target of taxane-based therapies, and stabilization of microtubules leads to mitotic arrest and apoptosis. Acetylation of α-tubulin has been widely used as an indicator of stabilized microtubules [15,25] and has been associated with improved therapeutic response and reduced recurrence in breast cancer [26]. In this study, inhibition of PAK4 enhanced docetaxel-induced α-tubulin acetylation and promoted microtubule stabilization, whereas phosphomimetic activation of PAK4 produced the opposite effect. These findings suggest that PAK4 phosphorylation is associated with reduced docetaxel-induced microtubule stabilization and provides a mechanistic link between adaptive kinase activation and reduced taxane sensitivity.

From a therapeutic perspective, our findings suggest that adaptive kinase activation represents a potential vulnerability for improving taxane efficacy. Taxane-based chemotherapy remains a cornerstone treatment for breast cancer but is frequently limited by dose-related toxicities [27]. In this study, pharmacological inhibition of PAK4 using LCH-7749944 enhanced docetaxel-induced cytotoxicity, apoptosis, and microtubule stabilization, indicating that targeting adaptive PAK4 activation may enhance cellular sensitivity to taxane treatment. Therefore, combination strategies involving PAK4 inhibition may represent a rational approach to overcome docetaxel resistance in breast cancer. Further studies are warranted to evaluate the therapeutic potential of clinically applicable PAK4 inhibitors.

Several limitations of this study should be acknowledged. First, although our results demonstrate that PAK4 phosphorylation regulates docetaxel-induced microtubule stabilization, the direct molecular link between PAK4 activation and microtubule regulation remains to be elucidated. Second, the upstream mechanisms underlying the relatively late activation of PAK4 phosphorylation after docetaxel exposure require further investigation. Third, the clinical relevance and therapeutic potential of targeting PAK4 require further validation in larger patient cohorts, patient-derived models, and in vivo studies.

## 5. Conclusions

Our study identifies PAK4 phosphorylation at S474 as an adaptive response induced by docetaxel treatment and demonstrates that inhibition of PAK4 enhances docetaxel sensitivity by promoting microtubule stabilization. These findings reveal a previously unrecognized adaptive signaling mechanism that attenuates docetaxel efficacy and highlight PAK4 signaling as a potential therapeutic target for improving docetaxel response in breast cancer.

## Data Availability

The original contributions presented in this study are included in the article and Supplementary Material. Further inquiries can be directed to the corresponding author.

## Supplementary Materials

The following supporting information can be downloaded at https://www.mdpi.com/article/10.3390/biomedicines14071631/s1: Supplementary Table S1. Protein identification and quantification information from quantitative proteomic analysis; Supplementary Table S2. Phosphorylation site identification and quantification information from phosphoproteomic analysis; Supplementary Figure S1. Quality control and reproducibility of proteomic and phosphoproteomic datasets; Supplementary Figure S2. Protein–protein interaction (PPI) network and functional module analysis of differentially phosphorylated proteins; Supplementary Figure S3. Association of PAK4 phosphorylation with docetaxel sensitivity across breast cancer cell lines; Figure S4. Overexpression of PAK4 WT and S474D mutant in MCF7 cells mediates docetaxel sensitivity.

## Abbreviations

The following abbreviations are used in this manuscript:

BC	Breast cancer
OS	Overall survival
pCR	Pathological complete response
DTX	Docetaxel
PAK4	p21-activated kinase 4
TCGA	The Cancer Genome Atlas
CPTAC	Clinical Proteomic Tumor Analysis Consortium
