# Supplementary material for: Phosphoproteomic Profiling Identifies PAK4 S474 Phosphorylation Affects Docetaxel Chemosensitivity via Modulation of Microtubule Stabilization in Breast Cancer
Source: Biomedicines. 2026 Jul 20;14(7):1631. doi: 10.3390/biomedicines14071631 (PMC13406789; doi:10.3390/biomedicines14071631)
Supplement: Supplementary file 1 [file biomedicines-14-01631-s001.zip › Supplementary Figure.pdf]

Supplementary Figures

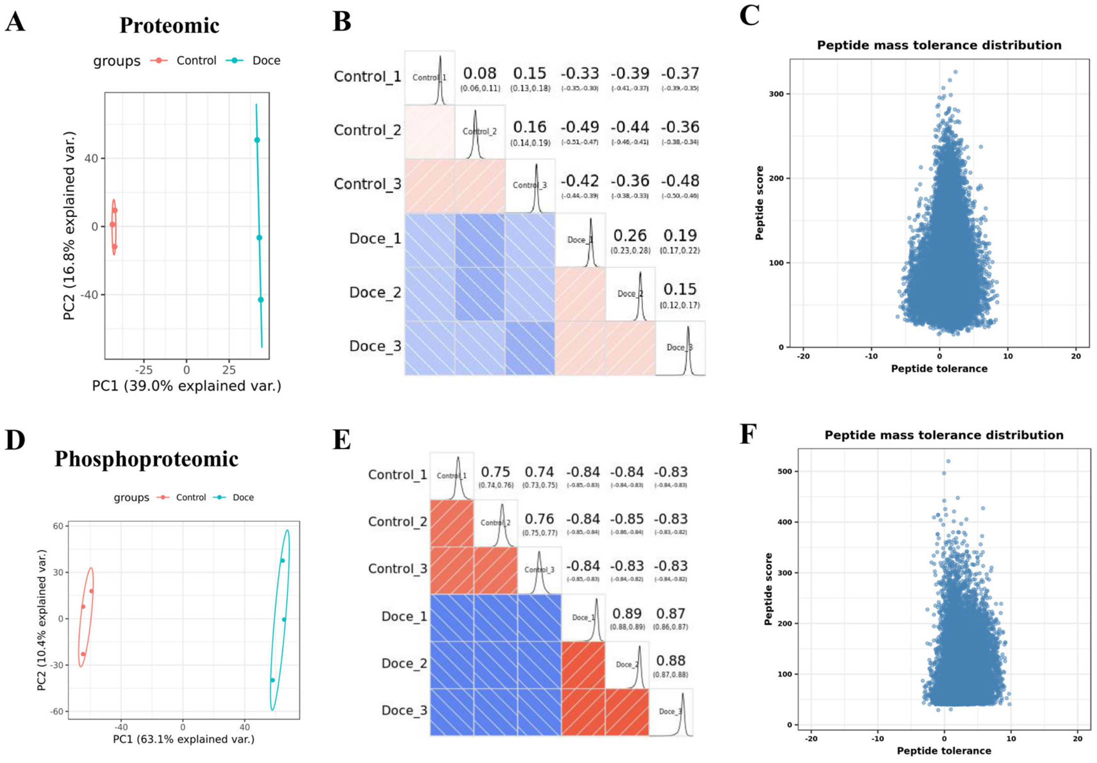

Figure S1. Quality control and reproducibility of proteomic and phosphoproteomic datasets. (A) Principal component analysis (PCA) showing separation between control and docetaxel-treated groups. (B) Pearson correlation matrix indicating high reproducibility within groups. (C) Peptide mass tolerance distribution demonstrating mass accuracy. (D) PCA of the phosphoproteome showing treatment-associated variance. (E) Pearson correlation heatmap of phosphopeptide quantification. (F) Phosphopeptide mass tolerance distribution.

Supplementary Figures

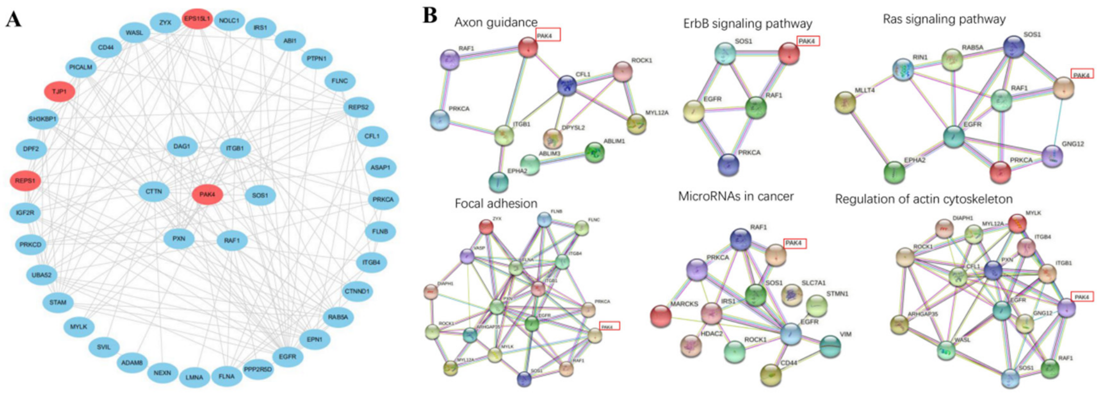

Figure S2. Protein–protein interaction (PPI) network and functional module analysis of differentially phosphorylated proteins. (A) Global PPI network, with upregulated proteins in red and downregulated proteins in blue. (B) Subnetworks centered on PAK4 and associated proteins across enriched pathways, including focal adhesion, regulation of actin cytoskeleton, ErbB signaling, Ras signaling, and axon guidance.

Supplementary Figures

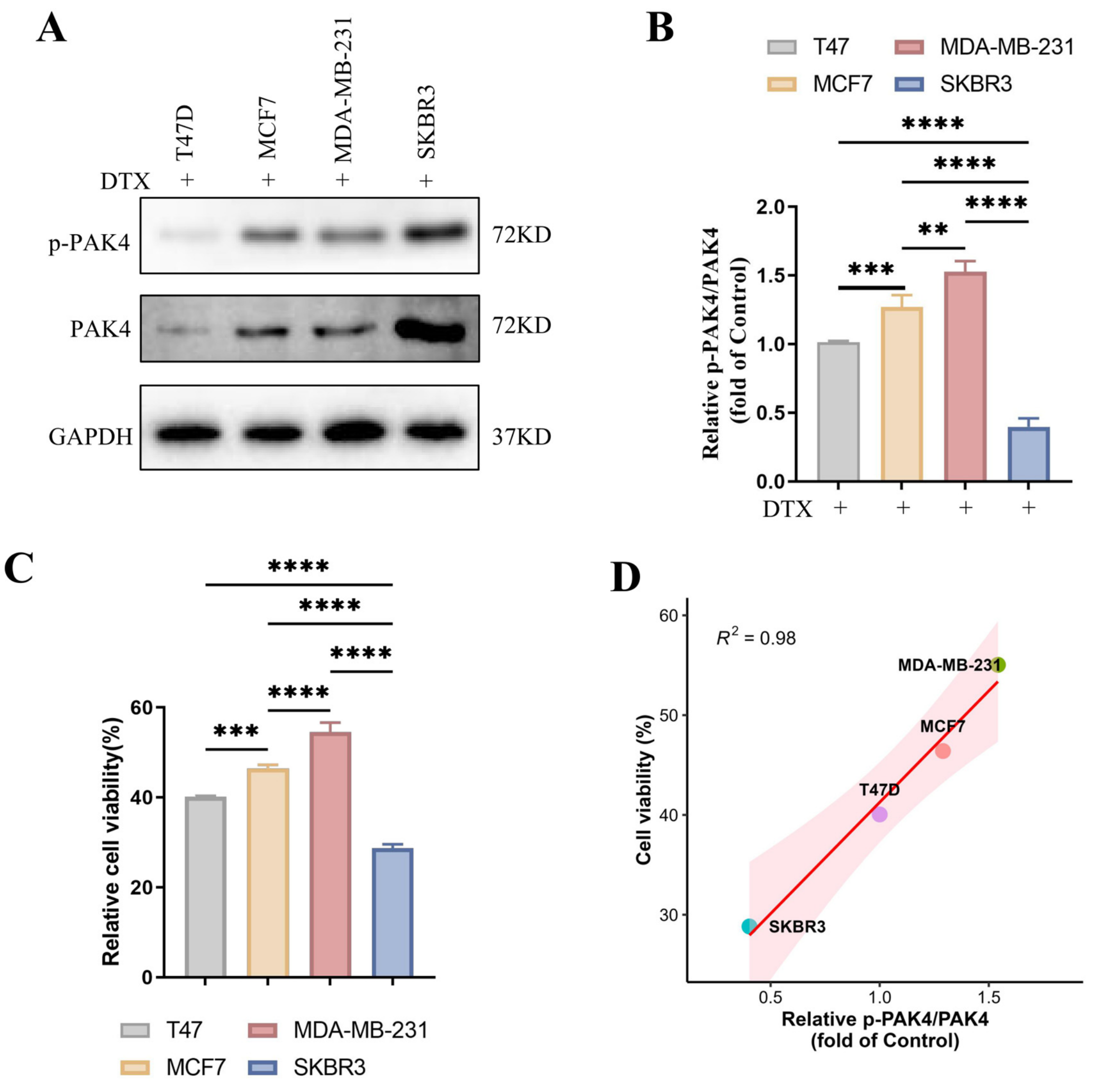

Figure S3. Association of PAK4 phosphorylation with docetaxel sensitivity across breast cancer cell lines. (A) Western blot analysis of p-PAK4 and PAK4 protein levels in T47D, MCF7, MDA-MB-231, and SKBR3 cells with docetaxel (DTX) treatment. (B) Quantification of the relative p-PAK4/PAK4 ratio in the four cell lines. (C) Cell viability of T47D, MCF7, MDA-MB-231, and SKBR3 cells treated with 50 ng/mL docetaxel, assessed by CCK-8 assay. (D) Scatter plot illustrating the linear trend between the relative p-PAK4/PAK4 levels and cell viability across four breast cancer cell lines. The red line represents the linear regression fit (R^2=0.98). Data are presented as mean ± SD. Statistical significance was determined by one-way ANOVA. **P < 0.01, ***P < 0.001, ****P < 0.0001.

Supplementary Figures

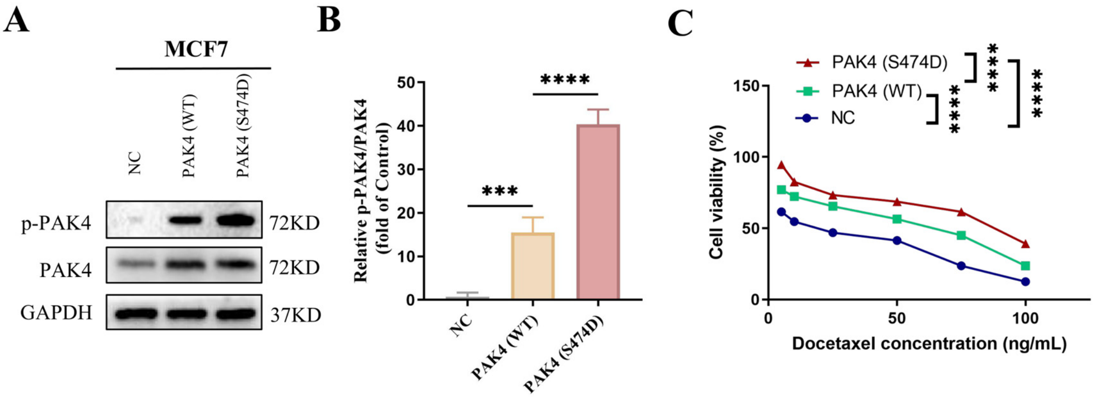

Figure S4. Overexpression of PAK4 WT and S474D mutant in MCF7 cells mediates docetaxel sensitivity. (A-B) Western blot analysis and quantitative evaluation of p-PAK4 and PAK4 expression in MCF7 cells transfected with control vector (NC), wild-type PAK4 (PAK4 WT), or phosphomimetic mutant (PAK4 S474D) plasmids. (C) Cell viability curves of MCF7 cells expressing pcDNA, PAK4 (WT), or PAK4 (S474D) under various concentrations of docetaxel treatment. Data are presented as mean ± SD. Statistical significance was determined by one-way ANOVA or two-way ANOVA . ***P < 0.001, ***P < 0.0001.
